# Association between serum Klotho concentration and all-cause and cardiovascular mortality among American individuals with hypertension

**DOI:** 10.3389/fcvm.2022.1013747

**Published:** 2022-11-15

**Authors:** Yuqin Yan, Jun Chen

**Affiliations:** Department of Cardiology, People’s Hospital of Shenzhen Baoan District, The 8th People’s Hospital of Shenzhen, Affiliated Baoan Hospital of Shenzhen, Southern Medical University, Shenzhen, China

**Keywords:** blood pressure, cardiovascular disease, chronic kidney disease, hypertension, Klotho, Centers for Disease Control and Prevention, National Health and Nutrition Examination Survey

## Abstract

**Background and aims:**

Evidence indicates that serum Klotho concentration is associated with mortality in patients with chronic kidney disease (CKD). However, evidence on this association among people with hypertension is scarce. Therefore, we aimed to examine the association between serum Klotho concentration and all-cause and cardiovascular mortality in American patients with hypertension.

**Methods and results:**

We included 6,778 participants with hypertension from the National Health and Nutrition Examination Survey (NHANES) 2007–2014. A Cox proportional hazard model was used to compute the hazard ratios (HRs) and 95% confidence intervals (CIs). The correlation between serum Klotho concentration and mortality was determined using restricted cubic spline and piecewise linear regression analyses. During 36,714 person-years of follow-up, 575 deaths were documented. Lower serum Klotho concentration was associated with increased all-cause mortality, but not cardiovascular mortality after multivariate adjustment. According to spline analysis, the correlation between serum Klotho concentration and all-cause mortality was non-linear (*P* < 0.001), and the threshold value was 574 pg/mL. The HR below the threshold point was 0.79 (95% CI: 0.67–0.93); no significant difference was found above the threshold point.

**Conclusion:**

Higher serum Klotho concentration was associated with lower all-cause mortality, but not cardiovascular mortality in patients with hypertension with or without chronic renal impairment.

## Introduction

Hypertension is a common disease that affects over 1 billion people globally (1). It is a public health burden with increasing prevalence and risk of adverse health outcomes, such as coronary heart disease, chronic heart failure, stroke, chronic kidney disease (CKD), and cognitive impairment. In addition, it is the leading cause of all-cause mortality and disability worldwide (2). Treatment of hypertension has been clinically demonstrated to reduce the risk of cardiovascular disease (CVD) outcomes (including stroke, myocardial infarction, heart failure, and death) and all-cause mortality (3, 4). However, current hypertension medications do not completely eradicate the clinical problems of high blood pressure (BP). Therefore, it is imperative to identify and intervene early in hypertensive patients at risk of death in order to reduce mortality rates.

Klotho was unexpectedly discovered in 1997; α-Klotho is the protein product of the Klotho gene, which was first identified as an aging suppressor (5, 6). There are three forms of the α-Klotho protein—membranous, soluble and secreted (7). However, the secreted Klotho isoform is not found in humans (7). Membrane Klotho functions as an obligatory fibroblast growth factor 23 (FGF23) coreceptor, facilitating FGF23-dependent urine phosphate excretion (8, 9). Soluble Klotho, the main functional form presents in the circulation, which functions as an endocrine and paracrine factor that affects multiple organs, such as kidney, brain, heart and lungs and endothelium (10–12).

Accumulating evidence indicates that Klotho gene polymorphisms and serum Klotho concentration are associated with the incidence and development of hypertension (13, 14). It is noteworthy that decreased serum Klotho concentrations are independent predictors of death and cardiovascular events in both hemodialysis and non-hemodialysis CKD patients (15, 16). Recent study findings have supported the hypothesis that serum Klotho concentration is inversely associated with all-cause mortality in adults (17, 18). According to the research, low circulating Klotho concentration may be a marker of mortality; however, the association between serum Klotho concentration and mortality in patients with hypertension is unclear. To address the gap in knowledge, we aimed to examine the association between serum Klotho concentration and all-cause and cardiovascular mortality in American patients with hypertension. And we hypothesized that Klotho concentrations would be associated with mortality and cardiovascular death in hypertensive patients, and that the mortality risk increases with decreasing Klotho concentration.

## Materials and methods

### Study population

The National Health and Nutrition Examination Survey (NHANES) of the United States is a stratified, multistage probability sample of people selected from the general population at random. Detailed technical information can be found in the Sample Design documents, available on the NHANES Survey Methods and Analytic Guidelines page^1^ (19–22). Participants undergo an in-depth interview and are subjected to medical and physiological assessment as well as laboratory tests such as serum, plasma, urine and DNA. The National Center for Health Statistics Research Ethics Review Board approved the survey protocol, and written informed consent was received from all participants (23). Data were continuously collected and released in 2-year cycles; the present study is a secondary analysis of these data. All processes were conducted in accordance with relevant standards and regulations as detailed in NHANES: plan and operations (24).

**FIGURE 1:**
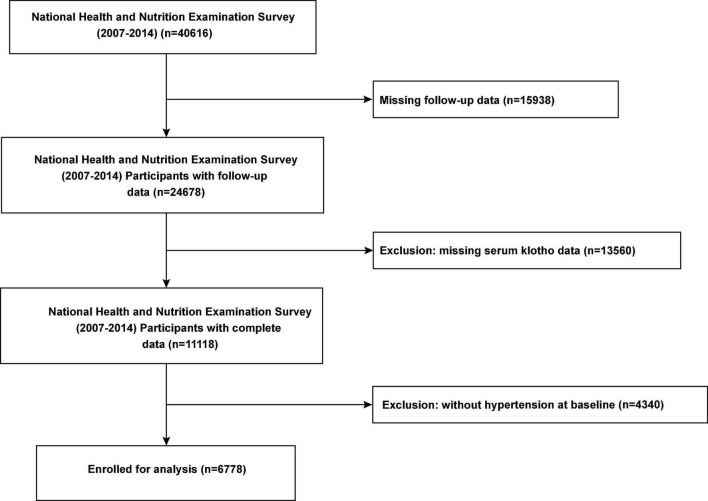
Flow chart of the study population.

For our investigation, we used publicly available de-identified data. Interview questionnaires and exam response rates are available to the public; more details are available on the official website.^2^ For the present study, we included data of all participants with hypertension who participated in the continuous NHANES cycles 2007–2008, 2009–2010, 2011–2012, and 2013–2014. After excluding individuals without follow-up data and information serum Klotho concentration, we included 6,778 participants with hypertension (Figure 1). This study was performed in accordance with the Strengthening the Reporting of Observational Studies in Epidemiology reporting guidelines.

### Hypertension definition

The BP of each patient were assessed after resting quietly in a sitting position for 5 min; three consecutive BP readings were recorded, and the mean of the 3 readings was calculated as the average BP. Participants were considered to have hypertension if the average systolic BP (SBP) was ≥140 mmHg, or the average diastolic BP (DBP) ≥90 mmHg, or they self-reported being previously diagnosed with hypertension by a physician, or taking antihypertensive medications (such as diuretics, angiotensin-converting enzyme inhibitors, angiotensin receptor blockers, calcium-channel blockers, and β-blockers).

### Exposure

All serum samples were flash frozen and maintained at −80°C until predetermined batches of samples were sent to the technicians for daily analysis. Serum Klotho concentration was quantified using a commercially available enzyme-linked immunoassay kit (IBL International, Hamburg, Germany) (25). Samples were tested in duplicate, and the average of the two values was used to obtain the final value. If >10% samples had duplicate results, the analyses were repeated. The sample analysis was repeated if the value of a quality control sample was not within two standard deviations (SDs) of the allocated value. The assay sensitivity was 6 pg/mL (26). There are no specific cut-points for Klotho. The reference range was evaluated in 114 samples from healthy donors and levels ranged from 285.8 to 1638.6 pg/mL (mean = 698.0 pg/mL) (26, 27).

### Other variable definitions

We included demographic variables including body mass index (BMI), medication use, age, sex, race, and education level as well as laboratory data as covariates. Laboratory data included levels of total cholesterol, high-density lipoprotein, triglycerides, alkaline phosphatase, creatinine, uric acid, blood urea nitrogen (BUN), fasting plasma glucose, hemoglobin A1c (HbA1c), and Klotho-related mineral metabolism markers (calcium, phosphate, and 25(OH) vitamin D). BMI was calculated as weight (in kilograms) divided by height (in meters squared). Diabetes was defined as a self-reported history of diabetes, use of oral hypoglycemic agents or insulin, fasting plasma glucose level ≥126 mg/dL, or HbA1c level ≥6.5%. CVD was defined as a history of coronary artery disease, angina, heart attack, or heart failure. Smoking status was classified as never smoker, former smoker, or current smoker. The estimated glomerular filtration rate (eGFR) was estimated using the CKDs Epidemiology Collaboration equation (28), CKD was defined as an eGFR of <60 mL/min/1.73 m^2^. As discussed later in the study, CKD was defined using eGFR of only one time point because the cross-sectional study did not measure the duration of reduced eGFR.

### All-cause and cardiovascular mortality

The main outcomes were all-cause and cardiovascular mortality. Participants were linked to the National Death Index until December 31, 2015 through a rigorous probability matching and death certificate review process. The cause of death was classified according to the International Classification of Diseases, Tenth Revision, codes. Cardiovascular mortality was defined according to the International Classification of Diseases, Tenth Revision, codes I00–I09, I11, I13, and I20–I51.

### Statistical analysis

Participants were divided into three groups according to their serum Klotho concentration, 500, 500–1,000, and >1,000 pg/mL, based on the restricted cubic spline model results. Continuous data are expressed as mean ± SD when normally distributed and as median (interquartile range) when not normally distributed. Categorical variables are expressed as percentages. For continuous variables, analysis of variance or the Kruskal–Wallis tests were used, while for categorical variables, the chi-square test or Fisher’s exact test was used.

We plotted cumulative Kaplan–Meier curves for all-cause and cardiovascular mortality during follow-up according to the predefined groups of serum Klotho concentration. The statistical significance of subgroup differences was evaluated using the log-rank test. Cox proportional hazard regression analysis was performed to estimate hazard ratios (HRs) and 95% confidence intervals (CIs) for associations between serum Klotho concentration and all-cause and cardiovascular mortality. We built three models to provide statistical inference. Confounders were selected based on a significant association with the outcomes of interest or a change in effect estimate of >10%. Model 1 included only serum Klotho concentrations. Model 2 included demographic variables (age, sex, race, education level), total cholesterol level, high-density lipoprotein-C level, HbA1c level, comorbidities (CVD, diabetes, and stroke), and use of medicines (hypotensive drugs, hypoglycemic drugs, lipid-lowering medication, and antiplatelet drugs). Model 3 included variables from model 2 plus BMI; smoking status; and levels of triglycerides, alkaline phosphatase, uric acid, BUN, creatinine, 25(OH) vitamin D, calcium, and phosphorus.

Restricted cubic spline models were built to detect any non-linear relationship between serum Klotho concentration and mortality. If non-linear relationships were identified, we used piecewise regression models to elucidate how the associations differed around the threshold point. The log-likelihood ratio test—comparing the one-line (non-segmented) regression model with the segmented regression model—was used to determine whether a threshold existed. The inflection point that connected the segments was based on the model demonstrating the maximum likelihood; it was determined using a two-step recursive method. In addition, we performed multiple imputation based on five replications, and a chained equation approach method was used in the R MI process to evaluate whether the use of indicator variables for missing data caused bias in our results.

Subgroup analyses were performed to test the robustness of our main findings. We tested for effect modification according to sex, age, smoking status, SBP, DBP, BMI, eGFR, and presence of diabetes and CVD. All analyses were conducted using R version 4.1.0 (R Foundation for Statistical Computing, Vienna, Austria), and a two-sided *P*-value of < 0.05 was considered statistically significant for all analyses, including interaction terms.

## Results

### Baseline characteristics

The baseline characteristics of the study participants according to serum Klotho concentration are shown in Table 1. The average (SD) age of the 6,778 participants was 60.9 (10.5) years; 49.5% participants were men. The mean (SD) Klotho concentration was 850 pg/mL (310) and 575 (8.5%) patients died after a median follow-up of 65 months. Participants with low serum Klotho concentration were more likely to be men, older, smokers, and drinkers and have low DBP. Participants with the lowest Klotho concentration were more likely to have diabetes and CVD and use hypotensive, hypoglycemic, and lipid-lowering medications. Laboratory tests revealed that participants with the highest Klotho concentration had reduced serum creatinine, BUN, uric acid, 25(OH) vitamin D levels and a low mortality rate. There were no observed differences in education level, SBP, calcium level, phosphorus level, and history of stroke between the groups.

**TABLE 1:** Characteristics of American hypertensive populations according to serum Klotho concentrations.

	Serum Klotho (100 pg/ml)				*P*-value
	
	Total	<5	5–10	>10	
Number	6778	485	4668	1625	
Age (years)	60.9 ± 10.5	62.7 ± 10.2	61.1 ± 10.5	59.8 ± 10.3	< 0.001
Sex, *n* (%)					< 0.001
Male	3355 (49.5)	238 (49.1)	2399 (51.4)	718 (44.2)	
Female	3423 (50.5)	247 (50.9)	2269 (48.6)	907 (55.8)	
Race, *n* (%)					< 0.001
Non-Hispanic white	3032 (44.7)	201 (41.4)	2218 (47.5)	613 (37.7)	
Non-Hispanic black	1630 (24.1)	150 (30.9)	988 (21.2)	492 (30.3)	
Hispanic	688 (10.2)	42 (8.7)	463 (9.9)	183 (11.3)	
Others	1428 (21.1)	92 (19.0)	999 (21.4)	337 (20.7)	
Education level, *n* (%)					0.47
Less than high school	2066 (30.5)	154 (31.8)	1417 (30.4)	495 (30.5)	
High school	1603 (23.7)	115 (23.7)	1127 (24.1)	361 (22.2)	
Above high school	3104 (45.8)	216 (44.5)	2119 (45.4)	769 (47.34)	
Others	5 (0.1)	0 (0.0)	5 (0.1)	0 (0.0)	
Smoking, *n* (%)					< 0.001
Never	3308 (48.8)	211 (43.6)	2221 (47.6)	876 (53.9)	
Former	2197 (32.4)	172 (35.5)	1558 (33.4)	467 (28.7)	
Current	1271 (18.7)	101 (20.9)	888 (19.0)	282 (17.4)	
Drinking, *n* (%)					< 0.001
No	1944 (28.7)	112 (23.1)	1285 (27.5)	547 (33.7)	
Yes	4407 (65.0)	338 (69.7)	3094 (66.3)	975 (60.0)	
Others	427 (6.3)	35 (7.2)	289 (6.2)	103 (6.3)	
BMI, kg/m^2^	30.8 ± 7.0	30.8 ± 6.9	30.8 ± 7.0	30.9 ± 7.2	0.84
SBP, mmHg	133.6 ± 19.1	134.2 ± 20.1	133.3 ± 19.2	134.1 ± 18.8	0.25
DBP, mmHg	72.5 ± 14.2	69.4 ± 14.5	72.4 ± 14.1	73.5 ± 14.1	< 0.001
**Laboratory findings**					
Calcium, mg/dl	9.4 ± 0.4	9.4 ± 0.4	9.4 ± 0.4	9.5 ± 0.4	0.26
Phosphorus, mg/dl	3.7 ± 0.6	3.8 ± 0.7	3.7 ± 0.6	3.7 ± 0.6	0.05
ALP, U/L	72.5 ± 24.8	71.1 ± 26.0	71.5 ± 24.1	76.0 ± 25.9	< 0.001
25(OH) vitamin D, nmol/l	65.9 ± 28.0	66.7 ± 28.6	66.4 ± 28.2	64.0 ± 27.2	0.008
Creatinine	1.0 ± 0.7	1.3 ± 1.5	1.0 ± 0.6	0.9 ± 0.4	< 0.001
UA, mg/dl	5.8 ± 1.5	6.1 ± 1.6	5.9 ± 1.5	5.5 ± 1.4	< 0.001
BUN	14.8 ± 6.8	17.2 ± 9.3	14.9 ± 6.7	13.8 ± 5.8	< 0.001
TC, mg/dl	194.8 ± 44.1	192.7 ± 47.5	194.6 ± 43.8	196.0 ± 43.9	
TG, mg/dl	140.0 (94.0—213.0)	142.0 (98.0–224.0)	142.0 (96.0–214.8)	133.0 (87.0–205.0)	< 0.001
HDL, mg/dl	51.9 ± 16.2	52.6 ± 18.0	51.7 ± 16.2	52.1 ± 15.9	0.43
HbA1c, %	6.1 ± 1.3	6.0 ± 1.0	6.1 ± 1.2	6.3 ± 1.6	< 0.001
FBG, mg/dl	114.0 ± 49.5	107.0 ± 35.0	111.7 ± 45.0	122.7 ± 62.9	< 0.001
Serum Klotho, 100 pg/ml	8.5 ± 3.1	4.3 ± 0.6	7.5 ± 1.3	12.7 ± 3.0	< 0.001
**Comorbidities**					
Diabetes					< 0.001
No	4569 (67.4)	300 (61.9)	3228 (69.2)	1041 (64.1)	
Yes	2209 (32.6)	185 (38.1)	1440 (30.9)	584 (35.9)	
Cardiovascular disease					0.004
No	5712 (84.3)	404 (83.3)	3896 (83.5)	1412 (86.9)	
Yes	1066 (15.7)	81 (16.7)	772 (16.5)	213 (13.1)	
Stroke					0.17
No	6300 (93.1)	442 (91.5)	4335 (93.0)	1523 (93.9)	
Yes	465 (6.9)	41 (8.5)	325 (7.0)	99 (6.1)	
**Medications, *n* (%)**					
Hypotensive drugs					0.002
No	2110 (31.1)	116 (23.9)	1473 (31.6)	521 (32.1)	
Yes	4668 (68.9)	369 (76.1)	3195 (68.4)	1104 (67.9)	
Hypoglycemic drugs					< 0.001
No	5213 (76.9)	338 (69.7)	3642 (78.0)	1233 (75.9)	
Yes	1565 (23.1)	147 (30.3)	1026 (22.0)	392 (24.1)	
Lipid-lowering medication					< 0.001
No	4109 (60.6)	263 (54.2)	2788 (59.7)	1058 (65.1)	
Yes	2669 (39.4)	222 (45.8)	1880 (40.3)	567 (34.9)	
Antiplatelet drugs					0.85
No	6225 (91.8)	445 (91.8)	4282 (91.7)	1498 (92.2)	
Yes	553 (8.2)	40 (8.3)	386 (8.3)	127 (7.8)	
All-cause mortality, *n* (%)					< 0.001
No	6203 (91.5)	421 (86.8)	4281 (91.7)	1501 (92.4)	
Yes	575 (8.5)	64 (13.2)	387 (8.3)	124 (7.6)	
Cardiovascular mortality, *n* (%)					0.72
No	6667 (98.4)	477 (98.4)	4588 (98.3)	1602 (98.6)	
Yes	111 (1.6)	8 (1.7)	80 (1.7)	23 (1.4)	

Data are expressed as mean ± SD, medians with interquartile ranges or percentages. BMI, body mass index; SBP, systolic blood pressure; DBP, diastolic blood pressure; 25(OH) vitamin D, 25-Hydroxyvitamin D; ALP, alkaline phosphatase; UA, uric acid; BUN, blood urea nitrogen; TC, total cholesterol; TG, triglyceride; HDL-C, high-density lipoprotein cholesterol; HbA1c, glycosylated hemoglobin; FBG, fasting blood glucose.

### Trends over time

There was a change in some variables over time with succeeding NHANES cycles. Between 2007–2008 and 2013–2014 NHANES cycles, smoking status; SBP; levels of creatinine, BUN, high-density lipoprotein, HbA1c, and fasting plasma glucose; comorbidities; and use of medication (excluding antiplatelet drugs) remained relatively stable. Despite the change in Klotho concentration between 2007–2008 and 2013–2014 NHANES cycles, the difference was not significant (Supplementary Table 1).

### Serum Klotho concentration and all-cause mortality

**FIGURE 2:**
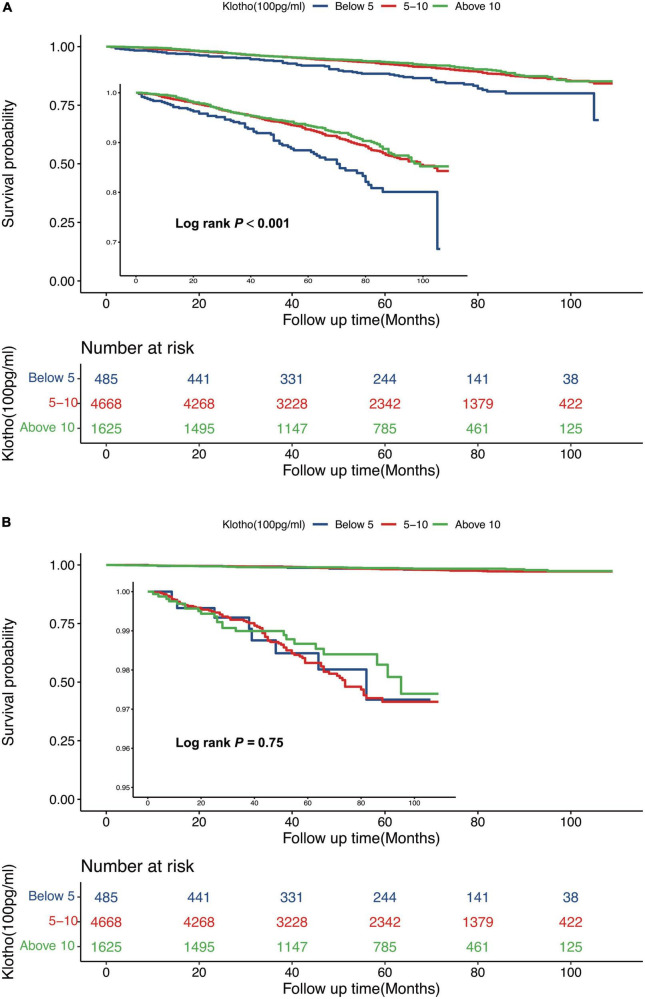
Kaplan-Meier (crude) survival curves, by serum Klotho concentrations, for all-cause mortality **(A)** and cardiovascular mortality **(B)**.

Serum Klotho concentration was inversely correlated with all-cause death; a 5% reduction in the risk of mortality was associated with each 100 pg/mL increase in serum Klotho concentration (*P* = 0.002). Even after multivariable adjustment, adverse associations persisted (Table 2). When serum Klotho concentration was analyzed as a categorical predictor using Cox regression estimates, compared to the overall risk of mortality at serum Klotho concentration <500 pg/mL, that at serum Klotho concentrations of 500–1,000 and >1,000 pg/mL decreased by 38% (95% CI: 0.12–0.52) and 42% (95% CI: 0.27–0.57), respectively (Table 2). Kaplan–Meier survival curves based on serum Klotho concentration are shown in Figure 2A. Participants with serum Klotho concentration <500 pg/ml had a significantly reduced overall survival compared to that in participants with higher Klotho concentrations (log-rank, *P* < 0.001).

**TABLE 2:** Hazard ratios (95% CIs) for the association of serum Klotho concentrations with all-cause mortality and cardiovascular mortality in American hypertensive populations.

	Model 1	Model 2	Model 3
**All-cause mortality**			
Serum Klotho level per 100 pg/ml increment	0.95 (0.93, 0.98) 0.002	0.97 (0.94, 1.00) 0.027	0.95 (0.93, 0.98) 0.003
<500 pg/ml	1.0	1.0	1.0
500–1,000 pg/ml	0.62 (0.48, 0.81) <0.001	0.72 (0.55, 0.94) 0.017	0.72 (0.54, 0.96) 0.025
>1,000 pg/ml	0.58 (0.43, 0.78) <0.001	0.69 (0.51, 0.95) 0.020	0.66 (0.47, 0.91) 0.011
*P* for trend	0.005	0.064	0.026
**Cardiovascular mortality**			
Serum Klotho level per 100 pg/ml increment	0.96 (0.90, 1.02) 0.21	0.97 (0.91, 1.04) 0.44	0.96 (0.89, 1.03) 0.27
<500 pg/ml	1.0	1.0	1.0
500–1,000 pg/ml	1.03 (0.50, 2.13) 0.94	1.04 (0.50, 2.17) 0.92	0.96 (0.45, 2.02) 0.90
>1,000 pg/ml	0.86 (0.38, 1.92) 0.71	0.93 (0.41, 2.11) 0.86	0.83 (0.36, 1.94) 0.67
*P* for trend	0.53	0.74	0.59

Data are presented as hazard ratios, 95% confidence intervals, and *P*-value. Model 1 adjusted for none. Model 2 adjusted for age, sex, race, education level, total cholesterol, high-density lipoprotein cholesterol, glycohemoglobin, comorbidities (cardiovascular disease, diabetes, and stroke), and medicine use (hypotensive drugs, hypoglycemic drugs, lipid-lowering medication, and antiplatelet drugs). Model 3 adjusted for age, sex, race, education level, smoking, body mass index, Calcium, Phosphorus, 25-Hydroxyvitamin D, alkaline phosphatase, uric acid, blood urea nitrogen, creatinine, total cholesterol, high-density lipoprotein cholesterol, triglyceride, glycohemoglobin, comorbidities (cardiovascular disease, diabetes, and stroke), and medicine use (hypotensive drugs, hypoglycemic drugs, lipid-lowering medication, and antiplatelet drugs).

**FIGURE 3:**
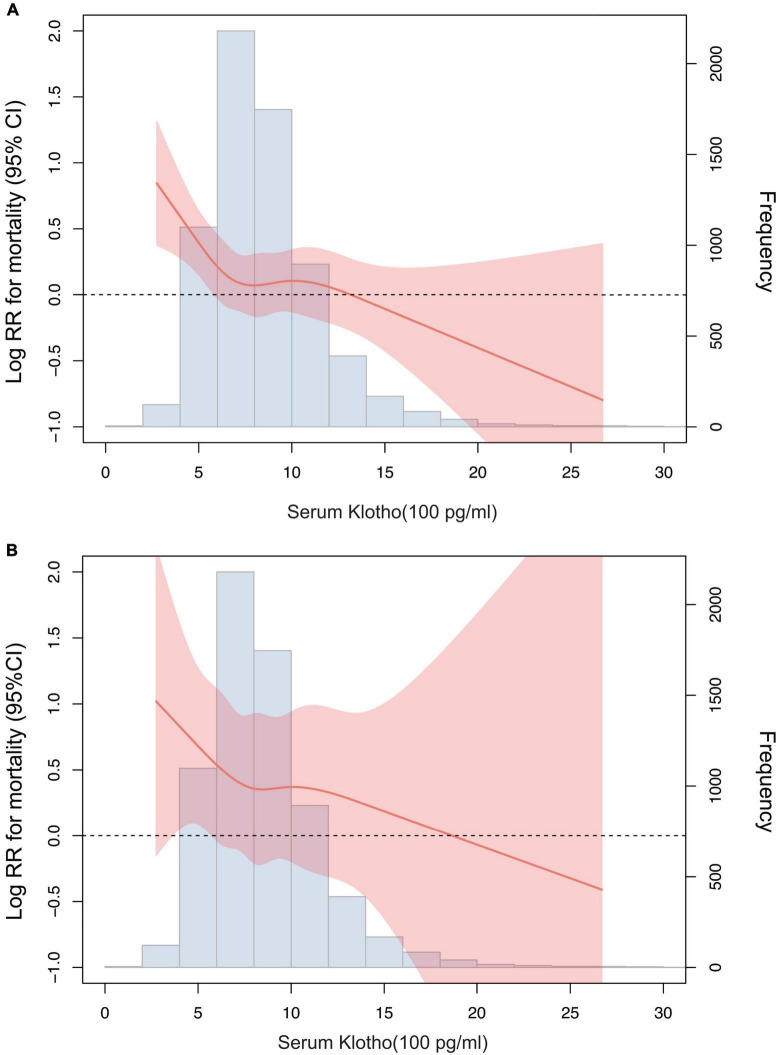
Association of serum Klotho concentrations with all-cause mortality **(A)** and cardiovascular mortality **(B)** using a restricted cubic spline regression model. Log RR, Log relative risk. Results were adjusted for age, sex, race, education level, smoking, body mass index, Calcium, Phosphorus, 25-Hydroxyvitamin D, alkaline phosphatase, uric acid, blood urea nitrogen, creatinine, total cholesterol, high-density lipoprotein cholesterol, triglyceride, glycohemoglobin, comorbidities (cardiovascular disease, diabetes, and stroke), and medicine use (hypotensive drugs, hypoglycemic drugs, lipid-lowering medication, and antiplatelet drugs).

According to the restricted cubic spline model, there was a high non-linear correlation between serum Klotho concentration and all-cause mortality (Figures 3A, *P* < 0.001). We calculated the threshold value of 574 pg/mL using a two-piecewise linear regression model. Serum Klotho concentration above the threshold point was correlated with a lower risk of all-cause mortality (HR: 0.79; 95% CI: 0.67–0.93), while that below the threshold point was not (Table 3; likelihood-ratio test, *P* = 0.026).

**TABLE 3:** The result of two-piecewise linear regression model between serum Klotho concentrations and all-cause mortality and cardiovascular mortality in American adults with hypertension.

	All-cause mortality	Cardiovascular mortality
Threshold value	5.74 (100 pg/mL)	4.65 (100 pg/mL)
<threshold value	0.79 (0.67, 0.93)	0.65 (0.33, 1.27)
≥threshold value	0.97 (0.97, 1.01)	0.97 (0.90, 1.05)
*P* for log likelihood ratio test	0.026	0.29

Data are presented as hazard ratios, 95% confidence intervals, and *P*-value. The two-piecewise linear regression models were adjusted for age, sex, race, education level, smoking, body mass index, Calcium, Phosphorus, 25-Hydroxyvitamin D, alkaline phosphatase, uric acid, blood urea nitrogen, creatinine, total cholesterol, high-density lipoprotein cholesterol, triglyceride, glycohemoglobin, comorbidities (cardiovascular disease, diabetes, and stroke), and medicine use (hypotensive drugs, hypoglycemic drugs, lipid-lowering medication, and antiplatelet drugs).

### Serum Klotho concentration and cardiovascular mortality

There was no significant association between serum Klotho concentration and cardiovascular mortality when serum Klotho concentration was analyzed as both continuous and categorical predictors using Cox regression estimates (Table 2 and Figure 2B). With a non-linear approach using penalized splines, we observed that cardiovascular mortality was not associated with high Klotho concentration (Figure 3B). The threshold value for cardiovascular mortality was 465 pg/mL; it was not statistically significant.

### Interactions

No significant interactions were observed between serum Klotho concentration and levels of phosphorus, calcium, and 25(OH) vitamin D and eGFR for both all-cause and cardiovascular mortality in the fully adjusted models.

### Subgroup analyses

**FIGURE 4:**
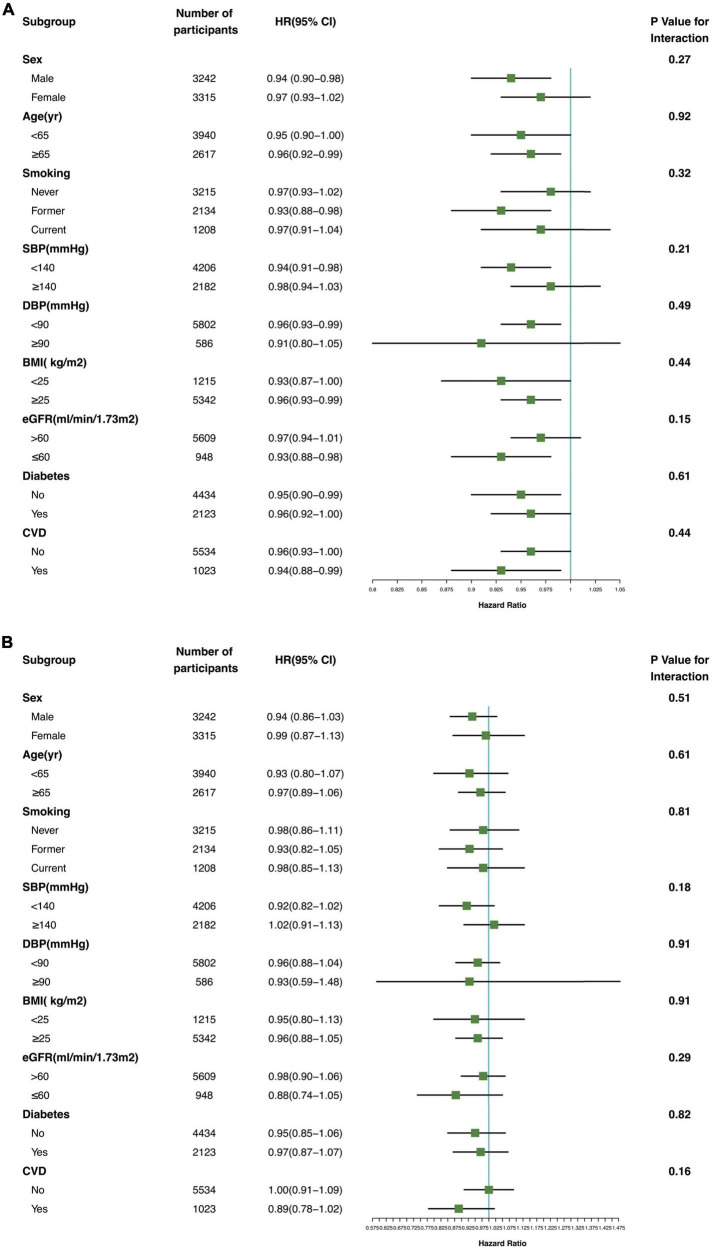
Association of serum Klotho concentrations with all-cause mortality **(A)** and cardiovascular mortality **(B)** in various subgroups. yr, years old; SBP systolic blood pressure; DBP, diastolic blood pressure; BMI, body mass index; eGFR, estimated glomerular filtration rate; CVD, cardiovascular disease.

The association between serum Klotho concentration and all-cause and cardiovascular mortality was consistent in subgroups according to sex, age, smoking status, DBP (≥90 vs. <90 mmHg), SBP (≥140 vs. <140 mmHg), BMI (≥25 vs. <25 kg/m^2^), eGFR (≥60 vs. <60 mL/min/1.73 m^2^), and presence or absence of diabetes and CVD (Figure 4; *P* for interaction >0.05).

### Sensitive analyses

The percentage of variables with missing data was 0–3%. Table 4 shows that by utilizing several imputed datasets, Cox regression analysis of serum Klotho concentration and all-cause and cardiovascular mortality produced comparable results to those using raw data.

**TABLE 4:** Cox proportional hazards analysis for all-cause and cardiovascular mortality according to serum Klotho concentrations in American hypertensive populations with imputed variables from multiple imputation.

	Model 1	Model 2	Model 3
**All-cause mortality**			
Serum Klotho level per 100 pg/ml increment	0.95 (0.93, 0.98) <0.001	0.97 (0.94, 1.0) 0.054	0.96 (0.94, 0.99) 0.002
<500 pg/ml	1.0	1.0	1.0
500–1,000 pg/ml	0.62 (0.48, 0.81) <0.001	0.69 (0.53, 0.90) 0.007	0.75 (0.57, 0.98) 0.033
>1,000 pg/ml	0.58 (0.43, 0.78) <0.001	0.67 (0.50, 0.91) 0.011	0.68 (0.50, 0.92) 0.014
*P* for trend	0.005	0.060	0.026
**Cardiovascular mortality**			
Serum Klotho level per 100 pg/ml increment	0.96 (0.90, 1.02) 0.20	0.97 (0.91, 1.04) 0.41	0.98 (0.92, 1.05) 0.55
<500 pg/ml	1.0	1.0	1.0
500–1,000 pg/ml	1.03 (0.50, 2.12) 0.50	1.05 (0.51, 2.19) 0.89	1.13 (0.54, 2.34) 0.54
>1,000 pg/ml	0.86 (0.38, 1.93) 0.72	0.94 (0.42, 2.11) 0.88	1.0 (0.44, 2.29) 0.99
*P* for trend	0.51	0.73	0.81

Data are presented as hazard ratios, 95% confidence intervals, and *P*-value. Model 1 adjusted for none. Model 2 adjusted for age, sex, race, education level, total cholesterol, high-density lipoprotein cholesterol, glycohemoglobin, and comorbidities (cardiovascular disease, diabetes, and stroke), and medicine use (hypotensive drugs, hypoglycemic drugs, lipid-lowering medication, and antiplatelet drugs). Model 3 adjusted for age, sex, race, education level, smoking, body mass index, Calcium, Phosphorus, 25-Hydroxyvitamin D, alkaline phosphatase, uric acid, blood urea nitrogen, creatinine, total cholesterol, high-density lipoprotein cholesterol, triglyceride, glycohemoglobin, comorbidities (cardiovascular disease, diabetes, and stroke), and medicine use (hypotensive drugs, hypoglycemic drugs, lipid-lowering medication, and antiplatelet drugs).

## Discussion

After 65 months of follow-up in a large cohort of American patients with hypertension from the NHANES, low serum Klotho concentration was associated with increased all-cause mortality, but not cardiovascular mortality. Moreover, there was a non-linear relationship between serum Klotho concentration and all-cause mortality. These findings were consistent in individuals with and without CKD.

Although a previous study reported that Klotho concentration correlated with lifespan in mice (5), studies on the association between circulating Klotho concentration and the risk of mortality in human populations are limited. A study on older, community-dwelling people demonstrated that after correcting for 25(OH)D, parathyroid hormone, and calcium levels and other potential confounders, plasma Klotho concentration was an independent predictor of mortality (18). Furthermore, Evangelos et al. indicated that Klotho deficiency was an independent predictor of cardiovascular and all-cause mortality in patients treated with chronic hemodialysis and the elderly population (29). According to a meta-analysis, low circulating soluble Klotho concentration was strongly related to increased all-cause mortality in patients with CKD (30). However, these studies have severe drawbacks, including the small size, inclusion of only populations with CKD or older age, and lack of a precise explanation for the actual relationship between Klotho concentration and mortality as well as the threshold value.

Our study showed that serum Klotho concentration was not associated with cardiovascular mortality in hypertensive population, in contrast to Christophe Marçais et al. (31), who found that Klotho concentration is associated with cardiovascular morbidity and mortality during hemodialysis. A major reason for the inconsistency was the study population. In Christophe Marçais’ study, the majority of the study population was composed of CKD patients receiving hemodialysis. CKD patients, particularly those receiving hemodialysis, have a higher risk of cardiovascular events like acute myocardial infarction, heart failure, and CVD death than normal individuals and hypertensive patients (32). The number of participants and the duration of follow-up may also be contributing factors. For the study by Christophe Marçais et al., 769 patients were recruited, and 238 were analyzed after 2 years of follow-up. Comparatively, our study analyzed 6,778 participants with a median follow-up of 65 months. Overall, our results were more robust and provided further evidence for the association between Klotho and cardiovascular mortality. Data from the Ludwigshafen Risk and Cardiovascular Health Study demonstrated that Klotho concentrations do not add predictive power to mortality risk assessment in patients with normal renal function (33). Nonetheless, even in patients with normal renal function (eGFR >60 mL/min/1.73 m^2^), low Klotho concentration was an independent risk factor for all-cause mortality in our analysis. The unadjusted and fully adjusted models revealed a non-linear relationship between serum Klotho concentration and all-cause mortality in our study. Moreover, in our study, the threshold value of serum Klotho concentration to predict all-cause mortality in patients with hypertension was 574 pg/mL, which has not been previously discussed.

Globally, hypertension is a highly prevalent condition and the main cause of CVD, stroke, CKD, and premature mortality (34). Many factors attribute to the death of hypertensive patients, such as lipid disorders, overweight-obesity, unhealthy lifestyle habits (e.g., smoking) (35). However, to the best of our knowledge, no previous study has evaluated the association between Klotho and mortality in individuals with hypertension. It has been shown that Klotho deficiency results in arterial stiffness and hypertension in mice, whereas Klotho supplementation prevents CVD from progressing in mice (36, 37). Serum Klotho deficiency is also associated with hypertension in humans. In the Health, Aging and Body Composition Study, low Klotho concentration was associated with a high risk of initial hypertension and high BP trajectories during follow-up (13).

Klotho is involved in the control of BP, through its influence in sodium handling, inflammatory cytokine activation, tissue cell infiltration and response to salt (38). By activating the non-canonical Wnt5a/RhoA pathway, Klotho deficiency increases salt sensitivity in patients with hypertension which leads to increased total peripheral resistance *via* vasoconstriction, therefore raising BP (39). Serum Klotho concentration is also independently associated with arterial stiffness (40); Klotho deficiency accelerates vascular aging and arterial stiffness by increasing autophagy and downregulating Sirtuin-1 (41, 42). Klotho can improve endothelial function by activating superoxide dismutase and endothelial nitric oxide synthase *via* the phosphoinositide 3-kinase/protein kinase B/endothelial nitric oxide synthase pathway (14). Increased peripheral resistance, vascular aging, and endothelial dysfunction all are factors that contribute to increased BP (43–45), and the higher the BP, the greater the risk of mortality (46).

The effects of Klotho on the health and survival of patients with hypertension can be mediated in several ways. According to a large body of research, Klotho appears to reduce oxidative stress, improve endothelial function, and provide vasoprotection at the cellular level. Furthermore, soluble Klotho has been shown to reduce fibrosis and block the epithelial–mesenchymal transition associated with fibrosis (47). Moreover, serum Klotho promotes endothelial nitric oxide generation, while also exerting systemic pleiotropic antioxidative, antithrombotic, and anti-inflammatory activities (30). According to research, FGF23 is associated with end-stage renal disease and acute kidney damage, severe infections and inflammation, and even cancer progression, which is considered a possible cause of death in people with CKD (48). It is believed that soluble Klotho protects against the effects of FGF23, which are exacerbated when serum Klotho concentration is reduced (48). And hypertensive patients with low Klotho concentration lose the above-mentioned protective mechanisms, predisposing them to mortality. As a result, lower serum Klotho concentration was associated with higher all-cause mortality in patients with hypertension.

Most critically, recent studies have demonstrated that Klotho supplementation protects mice from indoxyl sulfate-induced ventricular hypertrophy (49). According to Guo et al., injecting Klotho reduced cell mortality and remodeling (50). Additionally, Klotho therapy can protect against phosphotoxic insults, reduce oxidative stress, and reduce inflammation and fibrosis of the organs (38). Collectively, the therapeutic activity of Klotho in patients with CVD (including hypertension) represents a fascinating and promising perspective for the future.

The strengths of the current study include its relatively large sample size and the use of a nationally representative sample of the American population with hypertension, which facilitates the generalization of our findings. Furthermore, large demographic and comprehensive data may be utilized to account for a number of potential confounding factors, such as ethnicity; lifestyle traits; comorbidities; and laboratory data, such as calcium and phosphorus metabolism indicators and renal function assessment findings. Despite the encouraging results observed in this study, it has several potential limitations that should also be considered. First, our findings were restricted to populations with hypertension aged ≥40 years; thus, it is unclear whether the findings apply to people of various ages. However, hypertension usually develops in people aged >40 years. Second, since the FGF23/Klotho axis is associated with hypertension and CVD (51), we were unable to determine whether the clinical impact of Klotho on mortality was reliant on or independent of FGF23. Nonetheless, Klotho likely has FGF23-independent actions since circulating forms of Klotho without fibroblast growth factor receptors do not have a high affinity for FGF23 (41). Third, we defined CKD using creatinine levels measured at only one time point; however, previous studies based on national data or cohort studies also defined CKD based on the glomerular filtration rate measured at one time point (52). In addition, the proportion of patients with acute kidney injury among participants of a community-based research is expected to be negligible. Fourth, the likelihood of residual confounding effects from insufficient adjustment of mortality risk factors cannot be ruled out. Therefore, well-designed prospective studies are needed to assess and confirm these findings and further evaluate the biomarker and therapeutic potential of serum Klotho.

## Conclusion

Our findings indicate that low serum Klotho concentration was substantially correlated with a greater risk of all-cause death, but not cardiovascular mortality in a nationwide representative sample of American individuals with hypertension. Early diagnosis of low Klotho concentration (particularly <574 pg/mL) and appropriate intervention in patients with hypertension may be a possible target for preventing mortality. However, there is no definitive conclusion about Klotho’s normal concentration and no Klotho specific cut-points to be used as indicators of biological age, further studies to confirm these findings and explain the potential mechanisms for the observed associations are warranted.

## Data availability statement

The datasets presented in this study can be found in online repositories. NHANES data are publicly available at https://www.cdc.gov/nchs/nhanes/index.htm.

## Ethics statement

Ethical review and approval was not required for the study on human participants in accordance with the local legislation and institutional requirements. The patients/participants provided their written informed consent to participate in this study.

## Author contributions

YY conceived and designed the study and drafted reviewed and revised the manuscript. JC contributed to initial data analysis and interpretation. Both authors contributed to the article and approved the submitted version.

## Footnotes

1
https://wwwn.cdc.gov/nchs/nhanes/analyticguidelines.aspx#sample-design

2
https://www.cdc.gov/nchs/nhanes/index.htm

## Abbreviations

HR: hazard ratio
CI: confidence interval
BP: blood pressure
SBP: systolic blood pressure
DBP: diastolic blood pressure
FGF23: fibroblast growth factor 23
CKD: chronic kidney disease
CVD: cardiovascular disease
NHANES: National Health and Nutrition Examination Survey
SD: standard deviation
BMI: body mass index
BUN: blood urea nitrogen
HbA1c: hemoglobin A1c
eGFR: estimated glomerular filtration rate.
