# Supplementary material for: Association between serum Klotho concentration and all-cause and cardiovascular mortality among American individuals with hypertension
Source: Front Cardiovasc Med. 2022 Nov 15;9:1013747. doi: 10.3389/fcvm.2022.1013747 (PMC9705974; doi:10.3389/fcvm.2022.1013747)
Supplement: Supplementary file 1 [file Table_1.docx]

**TableS1 Baseline characteristics across 2007 to 2014.**

|  | Trends over 2007 to2014 | | | | *P*-value |
| --- | --- | --- | --- | --- | --- |
|  | **2007-2008** | **2009-2010** | **2011-2012** | **2013-2014** |  |
| Number | 1886 | 1731 | 1485 | 1676 |  |
| Age(years) | 61.5 ± 10.6 | 61.3 ± 10.5 | 60.2 ± 10.4 | 60.5± 10.4 | <0.001 |
| Sex, n (%) |  |  |  |  | 0.03 |
| Male | 943 (50.0) | 882 (51.0) | 752 (50.6) | 778 (46.4) |  |
| Female | 943 (50.0) | 849 (49.1) | 733 (49.4) | 898 (53.6) |  |
| Race, n (%) |  |  |  |  | <0.001 |
| Non-Hispanic White | 938 (49.7) | 835 (48.2) | 540 (36.4) | 719 (42.9) |  |
| Non-Hispanic Black | 421 (22.3) | 353 (20.4) | 461 (31.0) | 395 (23.6) |  |
| Hispanic | 201 (10.7) | 170 (9.8) | 168 (11.3) | 149 (8.9) |  |
| Others | 326 (17.3) | 373 (21.6) | 316 (21.3) | 413 (24.6) |  |
| Education level, n (%) |  |  |  |  | <0.001 |
| Less than high school | 654 (34.7) | 581 (33.6) | 408 (27.5) | 423 (25.2) |  |
| High school | 464 (24.6) | 410 (23.7) | 334 (22.5) | 395 (23.6) |  |
| Above high school | 767 (40.7) | 737 (42.6) | 743 (50.0) | 857 (51.1) |  |
| others | 1 (0.1) | 3 (0.2) | 0 (0.0) | 1 (0.1) |  |
| Smoking, n (%) |  |  |  |  | 0.63 |
| Never | 847 (50.5) | 736 (49.6) | 831 (48.0) | 894 (47.4) |  |
| Former | 303 (18.1) | 277 (18.7) | 327 (18.9) | 364 (19.3) |  |
| Current | 627 (33.3) | 573 (33.1) | 471 (31.7) | 526 (31.4) |  |
| Drinking, n (%) |  |  |  |  | <0.001 |
| No | 603 (32.0) | 483 (27.9) | 392 (26.4) | 466 (27.8) |  |
| Yes | 1193 (63.3) | 1114 (64.4) | 990 (66.7) | 1110 (66.2) |  |
| Others | 603 (32.0) | 483 (27.9) | 392 (26.4) | 466 (27.8) |  |
| BMI, kg/m^2^ | 30.4 ± 6.7 | 31.2 ± 6.8 | 30.8 ± 7.1 | 30.9 ± 7.4 | 0.007 |
| SBP, mmHg | 133.3 ± 18.7 | 133.2 ± 18.0 | 133.19 ± 20.0 | 134.3 ± 19.7 | 0.48 |
| DBP, mmHg | 71.9 ± 13.9 | 73.7 ± 14.2 | 71.7 ± 14.1 | 72.6 ± 14.4 | <0.001 |
| Laboratory findings |  |  |  |  |  |
| Calcium, mg/dl | 9.4 ± 0.4 | 9.5 ± 0.4 | 9.4 ± 0.39 | 9.47 ± 0.39 | <0.001 |
| Phosphorus, mg/dl | 3.7 ± 0.6 | 3.7 ± 0.6 | 3.7 ± 0.6 | 3.8 ± 0.6 | <0.001 |
| ALP, U/L | 74.0 ± 25.5 | 73.7 ± 23.9 | 72.1 ± 25.1 | 70.1 ± 24.3 | <0.001 |
| 25(OH) vitamin D, nmol/l | 61.2 ± 24.6 | 64.0 ± 26.1 | 67.9± 29.5 | 71.0 ± 30.8 | <0.001 |
| Creatinine | 0.96 ± 0.5 | 0.97 ± 0.7 | 0.99 ± 0.6 | 1.01 ± 0.8 | 0.17 |
| UA, mg/dl | 5.9 ± 1.5 | 5.8± 1.5 | 5.8 ± 1.5 | 5.7± 1.5 | 0.007 |
| BUN | 15.0 ± 6.8 | 14.7 ± 6.8 | 14.6 ± 6.4 | 15.0 ± 7.5 | 0.21 |
| TC | 199.0 ± 45.1 | 195.4 ± 44.1 | 192.5 ± 43.7 | 191.5 ± 42.8 | <0.001 |
| TG, mg/dl | 149.0 (100.0-221.0) | 140.0 (95.0-210.0) | 132.0 (89.0-206.8) | 136.0(91.0-208.0) | 0.005 |
| HDL, mg/dl | 51.5± 16.4 | 51.9 ± 16.8 | 51.9 ± 15.7 | 52.2 ± 16.0 | 0.62 |
| HbA1c, % | 6.1 ± 1.2 | 6.1 ± 1.2 | 6.2 ± 1.4 | 6.1 ± 1.3 | 0.24 |
| FBG，mg/dl | 113.3 ± 49.6 | 112.0 ± 45.5 | 114.3 ± 49.9 | 116.6 ± 52.9 | 0.05 |
| Serum Klotho, 100pg/ml | 8.4 ± 3.1 | 8.4 ± 3.2 | 8.7 ± 3.2 | 8.5 ± 2.9 | 0.01 |
| Comorbidities |  |  |  |  |  |
| Diabetes |  |  |  |  | 0.53 |
| No | 1266 (67.1) | 1186 (68.5) | 982 (66.1) | 1135 (67.7) |  |
| Yes | 620 (32.9) | 545 (31.5) | 503 (33.9) | 541 (32.3) |  |
| Cardiovascular disease |  |  |  |  | 0.07 |
| No | 1569 (83.2) | 1439 (83.1) | 1272 (85.7) | 1432 (85.4) |  |
| Yes | 317 (16.8) | 292 (16.9) | 213 (14.3) | 244 (14.6) |  |
| Stroke |  |  |  |  | 0.35 |
| No | 1734 (92.3) | 1613 (93.3) | 1383 (93.1) | 1570 (93.8) |  |
| Yes | 144 (7.7) | 116 (6.7) | 102 (6.9) | 103 (6.2) |  |
| Medications, n (%) |  |  |  |  |  |
| Hypotensive drugs |  |  |  |  | 0.46 |
| No | 614 (32.6) | 528 (30.5) | 459 (30.9) | 509 (30.4) |  |
| Yes | 1272 (67.4) | 1203 (69.5) | 1026 (69.1) | 1167 (69.6) |  |
| Hypoglycemic drugs |  |  |  |  | 0.41 |
| No | 1468 (77.8) | 1343 (77.5) | 1128 (76.0) | 1274 (76.0) |  |
| Yes | 418 (22.2) | 388 (22.4) | 357 (24.0) | 402 (24.0) |  |
| Lipid-lowering medication |  |  |  |  | 0.24 |
| No | 1177 (62.4) | 1044 (60.3) | 897 (60.4) | 991 (59.1) |  |
| Yes | 709 (37.6) | 687 (39.7) | 588 (39.6) | 685 (40.9) |  |
| Antiplatelet drugs |  |  |  |  | 0.002 |
| No | 1727 (91.6) | 1558 (90.0) | 1390 (93.6) | 1550 (92.5) |  |
| Yes | 159 (8.4) | 173 (1.0) | 95 (6.4) | 126 (7.5) |  |
| All-cause mortality, n (%) |  |  |  |  | <0.001 |
| No | 1589 (84.3) | 1579 (91.2) | 1408 (94.8) | 1627 (97.1) |  |
| Yes | 297 (15.8) | 152 (8.8) | 77 (5.2) | 49 (2.9) |  |
| Cardiovascular mortality |  |  |  |  |  |
| No | 1840 (97.6) | 1693 (97.8) | 1469 (98.9) | 1665 (99.3) | <0.001 |
| Yes | 46 (2.4) | 38 (2.2) | 16 (1.1) | 11 (0.7) |  |

Notes: Data are expressed as mean ± SD, medians with interquartile ranges or percentage.

Abbreviations: BMI, body mass index; SBP, systolic blood pressure; DBP, diastolic blood pressure; 25(OH) vitamin D,25-Hydroxyvitamin D; ALP, alkaline phosphatase; UA, uric acid; BUN, blood urea nitrogen; TC, total cholesterol; TG, triglyceride; HDL-C, high-density lipoprotein cholesterol; HbA1c, glycosylated hemoglobin; FBG, fasting blood glucose.
